# Decreased Frequency and Improved Outcomes in Invasive Aspergillosis Caused by *Aspergillus terreus* After the Introduction of Anti-Mold Azole Agents: A 30-Year Study at a Tertiary Cancer Center

**DOI:** 10.3390/jof11020119

**Published:** 2025-02-05

**Authors:** Ray Y. Hachem, Hiba Dagher, Anne-Marie Chaftari, Ying Jiang, Andrea Haddad, Saliba Wehbe, Jishna Shrestha, Robin Sherchan, Peter Lamie, Jennifer Makhoul, Patrick Chaftari, Issam I. Raad

**Affiliations:** 1 Department of Infectious Diseases, Infection Control and Employee Health, The University of Texas MD Anderson Cancer Center, Houston, TX 77030, USA; hrdagher@mdanderson.org (H.D.); achaftari@mdanderson.org (A.-M.C.); yijiang@mdanderson.org (Y.J.); ajhaddad@mdanderson.org (A.H.); swehbe@mdanderson.org (S.W.); jshrestha@mdanderson.org (J.S.); robin.sherchan@bcm.edu (R.S.); pmlamie@mdanderson.org (P.L.); jmakhoul@mdanderson.org (J.M.); iraad@mdanderson.org (I.I.R.); 2 Department of Emergency Medicine, The University of Texas MD Anderson Cancer Center, Houston, TX 77030, USA; pchaftari@mdanderson.org

**Keywords:** *Aspergillus terreus*, antifungal

## Abstract

Invasive aspergillosis (IA) is a significant cause of morbidity and mortality in patients with hematological malignancy (HM) and hematopoietic stem cell transplant (HSCT) recipients. *Aspergillus terreus* is associated with worse outcomes than non-*terreus Aspergillus* species. Since the introduction of anti-mold azoles in 2002, there have been limited data on the etiology of IA. We retrospectively compared characteristics, antifungal treatments, and outcomes between patients with HM or HSCT infected with *A. terreus* and those with non-*terreus Aspergillus* between July 1993 and July 2023. We also examined trends over time in rates of *A. terreus* and outcomes of this infection. A total of 699 patients with culture-documented IA were analyzed, 537 with non-*terreus* species and 162 with *A. terreus*. Types of underlying malignancy, neutropenia, graft-versus-host disease, and anti-mold prophylaxis were similar between the groups. ICU stays and mechanical ventilation were more common among patients with *A. terreus* (*p* = 0.002 and 0.003, respectively). The rate of *A. terreus* decreased significantly from 35.9% during 1993–2003 to 11.2% during 2004–2013 and 16.7% during 2014–2023 (*p* < 0.0001 each). IA caused by *A. terreus* showed significant improvements in response to therapy and in overall and IA-associated mortality in the last two decades compared to the first (*p* < 0.0001). In conclusion, the increased use of anti-mold azoles after 2003 improved outcomes for HM patients with IA caused by *A. terreus*.

## 1. Introduction

Invasive aspergillosis (IA) is a severe infection that predominantly affects immunocompromised patients, particularly those with hematological malignancies (HMs) and recipients of hematopoietic stem cell transplant (HSCT) [1,2]. The infection occurs via the inhalation of *Aspergillus conidia*, which is ubiquitous in the environment, including soil, plants, household dust, and building materials [3]. In susceptible hosts, these filamentous fungi can lead to invasive pulmonary infections or disseminate to extrapulmonary sites such as the skin, brain, or bones [4]. Among the various species capable of causing IA, *Aspergillus fumigatus* is the most encountered pathogen, accounting for more than 60% of all *Aspergillus* infections [5]; however, in the last several decades, there has been a notable shift in the epidemiological landscape of *Aspergillus* infections, with a growing incidence of non-*fumigatus* species, such as *Aspergillus terreus*, *Aspergillus flavus*, and *Aspergillus nidulans* [6]. This observation was facilitated by advancements in the detection and identification of *Aspergillus* species, including molecular diagnosis and improved non-culture-based methods [7].

*A. terreus* stands out among *Aspergillus* species primarily due to its resistance to amphotericin B and having a high frequency of dissemination, a characteristic rarely observed in species such as *fumigatus* [8,9,10]. This resistance is attributable to the expression of enzymes such as superoxide dismutase and catalase, which increase the oxidative stress response of *A. terreus*, thereby conferring protection against the damage caused by amphotericin B [11]. This intrinsic resistance significantly limits therapeutic options, and although alternatives such as triazoles have been used successfully for treatment, the overall prognosis for *A. terreus* infections remains poor [12,13]. Additionally, *A. terreus* is more prone to causing disseminated infections compared to other *Aspergillus* species, further complicating treatment [14]. These factors and others make *A. terreus* one of the most virulent species in the *Aspergillus* genus [15].

From an epidemiological perspective, *A. terreus* accounts for only a minority of IA cases, between 3% and 12.5% [15,16]; however, higher infection rates have been reported in certain geographic regions, such as Houston, Texas, in the United States and Innsbruck, Tyrol, in Austria [14]. The extensive use of triazoles for prophylaxis over the past 2 decades may have reduced the incidence of *A. terreus* infection in one center [14]. However, the long-term epidemiological trends of the pathogen remain underexplored. After the first introduction of voriconazole as an effective anti-mold azole for clinical use in the US on 24 May 2002, there have been limited data available to assess any change in the etiology of culture-documented IA.

Hence, the aim of this study was to compare the prevalence and outcomes of *A. terreus* IA infections with those of non-*terreus* IA infections after the introduction of effective anti-mold azoles (such as voriconazole and posaconazole) at our institution in 2003. To this end, we conducted a retrospective review of a large cohort of HM patients and HSCT recipients treated for definite or probable IA over the past three decades (1993–2023).

## 2. Materials and Methods

### 2.1. Patient Selection and Data Collected

We conducted a retrospective single-center study on patients with HM or HSCT within the preceding year who developed IA treated with antifungals at our cancer center between July 1993 and July 2023 with a follow-up period of 12 weeks. Cases were selected from the infection control database by searching microbiology records for all patients with proven or probable IA as defined below. We compared baseline characteristics, antifungal prophylaxis, and antifungal treatment between patients infected with *A. terreus* and those infected with non-*terreus Aspergillus*. We also examined the rate of *A. terreus* across 3 decades: 1993–2003, 2004–2013, and 2014–2023. The study was approved by the institutional review board; due to the retrospective nature of the study, a waiver for informed consent was granted.

### 2.2. Definition of IA

IA was required to be either proven or probable according to the joint guidelines of the European Organisation for Research and Treatment of Cancer and the Mycoses Study Group of the National Institute of Allergy and Infectious Diseases. Proven IA was defined by evidence of filamentous fungi plus associated tissue damage shown on histopathology, cytopathology, or the recovery of *Aspergillus* species from a specimen obtained by sterile needle aspiration or biopsy.

Probable IA includes mycological evidence of *Aspergillus* species with one of the following: (1) cytology and/or culture indicating the presence of *Aspergillus* species in a lower respiratory tract specimen; (2) a serum galactomannan antigen index of 0.5–1.0 for 2 consecutive findings or >1.0 once, or an index of >0.8 in bronchoalveolar lavage, provided that clinical/radiological abnormality and host factor criteria were met, such as the receipt of HSCT in the context of neutropenia lasting >14 days or the receipt of corticosteroids equivalent to >0.3 mg/kg prednisone for >3 consecutive weeks in the 60 days preceding the diagnosis of IA.

### 2.3. Statistical Methods

Chi-square or Fisher exact tests were used to compare categorical variables between groups, as appropriate. The Wilcoxon rank-sum test was used to compare continuous variables between groups. Trend analyses over the course of the 3 decades were performed for the rate of *A. terreus* infection among IA cases, as well as for the characteristics, treatments, and outcomes among patients infected with *A. terreus*, using the Cochran–Armitage trend test for categorical variables and the Jonckheere–Terpstra test for non-parametric continuous variables. Multivariate logistic regression analyses were performed to identify the independent predictors of the outcomes, including the independent effects of *A. terreus* infections. All the tests were 2-sided with a significance level of 0.05. The statistical analyses were performed using SAS version 9.4 (SAS Institute Inc., Cary, NC, USA).

## 3. Results

We identified 760 patients with IA during the study period between July 1993 and October 2023, of whom 537 patients were infected with identified *Aspergillus* species other than *terreus*, 162 patients were infected with *A. terreus* (alone or mixed with other *Aspergillus* species), and 61 patients were infected with unidentified *Aspergillus* species. The 699 cases with identified species were included in the analysis.

### 3.1. Terreus Versus Non-Terreus Infections

Patients with *A. terreus* infections were similar to those with non-*terreus* infections in most demographic and clinical characteristics, including the type of HM, HSCT history within 1 year before IA, and neutropenia status at IA onset, except that patients with *A. terreus* were a few years younger (median age: 53 vs. 56 years, *p* = 0.034) (Table 1).

Regarding infection locations, patients with *A. terreus* were more likely to have invasive pulmonary infections (89% vs. 82%, *p* = 0.029) and less likely to have sinus infections (1% vs. 6%, *p* = 0.01) compared to those infected with other species (Table 1). For treatment, patients with *A. terreus* were more likely to have received antifungal prophylaxis (74% vs. 62%, *p* = 0.007) and to receive amphotericin B–containing primary therapy (65% vs. 51%, *p* = 0.002) but less likely to receive anti-mold azole-containing primary therapy (38% vs. 58%, *p* < 0.0001) (Table 2). In addition, infections with *A. terreus* were more likely to lead to an intensive care unit (ICU) stay (51% vs. 37%, *p* = 0.002) and mechanical ventilation (41% vs. 28%, *p* = 0.003).

In univariate analysis, *A. terreus* infections showed significantly worse outcomes, including final response to therapy (30% vs. 43%, *p* = 0.003), all-cause 42-day mortality (51% vs. 39%, *p* = 0.009), IA-associated 42-day mortality (43% vs. 29%, *p* = 0.001), all-cause 84-day mortality (61% vs. 49%, *p* = 0.006), and IA-associated 84-day mortality (49% vs. 36%, *p* = 0.003) (Table 2).

### 3.2. Changes in A. terreus Infections over Time

**Figure 1:**
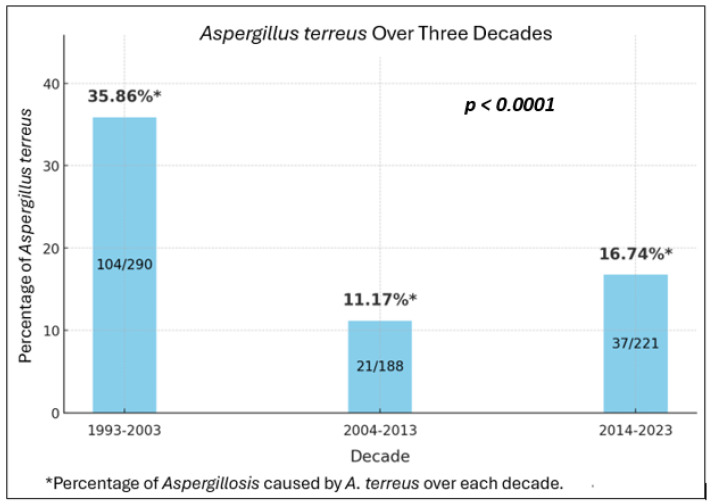
The percentages of *Aspergillus Terreus* over three decades.

We conducted trend analysis over time on the rate of *A. terreus* infection among IA cases, as well as characteristics and outcomes among patients with this infection. First, we found that the rate of *A. terreus* among IA cases significantly decreased in the last two decades compared to the first decade, from 35.9% during 1993–2003 to 11.2% during 2004–2013 and 16.7% during 2014–2023 (Figure 1).

Among patients with *A. terreus*, we found a significant increase in age, a significant decrease in the number of patients with leukemia, and a significant increase in the number of patients with lymphoma over time (Table 3). The rates of neutropenia, ICU stays, and mechanical ventilation were similar between all three decades. In the last two decades, patients who were diagnosed with *A. terreus* were less likely to have received antifungal prophylaxis and anti-mold prophylaxis than in the first decade. Similarly, patients in the last two decades were less likely to have been treated with an amphotericin B–containing regimen and more likely to have been treated with azole- and echinocandin-containing regimens (Table 3). Regarding outcomes, there was a significant improvement in the final response to therapy, with a higher rate of successes, and a decrease in 42- and 84-day overall mortality, as well as IA-associated mortality, in the last two decades compared to the first decade.

## 4. Discussion

Our findings indicate that over the last two decades, IA due to *Aspergillus terreus* has decreased. In addition, the final response to therapy significantly improved while mortality decreased over time in association with the introduction and increased therapeutic use of anti-mold azoles.

Invasive aspergillosis (IA) is a major cause of morbidity and mortality in immunocompromised patients [1,2]. These effects are especially pronounced in those patients infected with *A. terreus*, which is intrinsically resistant to amphotericin B therapy. In a 2002 trial, the mortality rate in patients with HM with invasive fungal infections ranged from 30% to 60%, particularly those with HSCT and acute leukemia [17].

Early diagnosis or prophylaxis may prevent these infections and improve outcomes. Because the diagnosis of fungal infection is often delayed, prophylaxis is commonly used. In the early 1990s, antifungal prophylaxis with fluconazole was helpful in reducing mortality and morbidity among recipients of HSCTs by decreasing the risk of invasive candidiasis [18]. However, fluconazole does not protect against mold infections, which is common seen in HSCT after engraftment in patients with graft-versus-host disease and in patients with leukemia after induction chemotherapy. Therefore, in patients with acute myelogenous leukemia (AML) or myelodysplastic syndrome (MDS) and those undergoing HSCT, prophylaxis with an anti-mold azole such as posaconazole or voriconazole may reduce the incidence of mold infections and improve overall survival. Cornely et al. reported that in patients undergoing chemotherapy for AML or MDS, posaconazole prevented invasive fungal infections more effectively than fluconazole or itraconazole and improved overall survival [19]. Similarly, in high-risk patients who had undergone HSCT, when posaconazole was compared to fluconazole in a randomized, double-blinded trial, posaconazole was superior in preventing IA and reducing the rate of death attributable to invasive fungal infections [20].

By the same premise, in the current study, the rate of *A. terreus* infection among IA cases was significantly reduced following the introduction of azole anti-mold agents (such as posaconazole and voriconazole) administered prophylactically and therapeutically during 2003–2023 (Figure 1). Of note, Torres et al. reported that during 1988–2001, there was a predominance of more resistant, non-*fumigatus* species such *A. terreus* among cancer patients [21], which is consistent with our finding of a high frequency of *A. terreus* (32%) during 1993–2003 (Figure 1).

Our group has previously published data from patients infected with IA caused by *A. terreus* from 1993 to 2012, demonstrating that IA due to *A. terreus* was associated with poor outcomes and higher mortality [12]. Similarly, our current results show that IA caused by *A. terreus* has significantly worse clinical outcomes and higher mortality compared to those caused by other *Aspergillus* species. However, after anti-mold azole agents such as voriconazole, posaconazole, and isavuconazole were introduced at our center after 2003, outcomes significantly improved over time in terms of response to therapy, as well as overall and IA-related mortality at 42 days and at 84 days.

Moreover, the management of IA over the last two decades has changed as treatment approaches have shifted towards azole-based therapies. In the second decade, patients with *A. terreus* were more likely to have received therapy with anti-mold active azole regimens and less likely to have received amphotericin B–containing regimens compared to those treated during the preceding decade (1993–2003), when the new anti-mold azoles (voriconazole, posaconazole, and isavuconazole) were not available. This change in therapeutic strategy has contributed to the improved outcomes observed at our institution over the last two decades despite the similar acuity of cases with *A. terreus*, as evidenced by the similarities in the underlying hematologic malignancy, neutropenic status, rates of ICU stay, and mechanical ventilation across the three decades. These findings demonstrate the evolving epidemiology of *A. terreus* infections in a tertiary cancer center, particularly in the context of azole prophylaxis, and are vital in improving antifungal stewardship practices and optimizing the management of IA in high-risk populations. Thus, this study will provide a good resource for clinicians dealing with invasive Aspergillosis in this patient population.

This study has several limitations, including its retrospective nature, as well as the follow-up and assessment, which were not controlled in this complex patient population. Furthermore, the choice of antifungal therapy and the decisions pertaining to the management of IA did not follow a protocol or algorithm and were determined according to each physician’s discretion.

In conclusion, the rate of IA due to *A. terreus* decreased significantly with the introduction of anti-mold active azole agents after 2003. Patients with *A. terreus* were more likely to have received therapy with anti-mold azole-containing and echinocandin-containing regimens after 2003 and less likely to have received amphotericin B–containing regimens compared to those treated during the earlier decade (1993–2003). This change in approach likely contributed to the improved outcomes observed in the last two decades (2004–2023) in patients with IA due to *A. terreus*.

## Figures and Tables

**Table 1:** Comparison of characteristics of patients with invasive aspergillosis with and without *Aspergillus terreus*.

Variables	Other *Aspergillus* Species	*A. terreus*	*p*-Value
	(N = 537)	(N = 162)	
IA diagnosis period			<0.0001
1993–2003	186/536 (35)	104 (64)	
2004–2013	167/536 (31)	21 (13)	
2014–2023	183/536 (34)	37 (23)	
Age (years), median (IQR)	56 (43–67)	53 (39–64)	0.034
Sex, male, n (%)	329 (61)	102 (63)	0.70
Diagnosis of IA, n (%)			0.074
Definite IA	160/529 (30)	37/161 (23)	
Probable IA	369/529 (70)	124/161 (77)	
Type of IA, n (%)			
Invasive pulmonary infection	438 (82)	144 (89)	0.029
Disseminated infection	41 (8)	7 (4)	0.14
Localized infection	32 (6)	8 (5)	0.62
Sinus infection	34 (6)	2 (1)	0.01
Type of hematological malignancy, n (%)			
Leukemia	362/536 (68)	121 (75)	0.08
Lymphoma	121/536 (23)	26 (16)	0.07
Myeloma	32/536 (6)	9 (6)	0.84
Transplant within 1 year before IA, n (%)	193/533 (36)	57/160 (36)	0.89
Type of transplant within 1 year before IA, n (%)			0.32
Autologous	34/187 (18)	7/56 (13)	
Allogeneic	153/187 (82)	49/56 (88)	
Graft-versus-host disease, n (%)	109/532 (20)	37/159 (23)	0.45
Neutropenia (ANC < 500) within 7 days of IA onset, n (%)	227/523 (43)	74/159 (47)	0.49

Abbreviations: ANC, absolute neutrophil count per µL. IA, invasive aspergillosis. IQR, interquartile range.

**Table 2:** Comparison of prophylaxis, treatments, and outcomes in patients with invasive aspergillosis with and without *Aspergillus terreus*.

Variables	Other *Aspergillus* Species	*A. terreus*	*p*-Value
	(N = 537)	(N = 162)	
Antifungal prophylaxis, n (%)	332/533 (62)	119/161 (74)	0.007
Primary antifungal therapy, n (%)			
Amphotericin B–containing regimen	276 (51)	106 (65)	0.002
Echinocandin-containing regimen	139 (26)	37 (23)	0.43
Azole-containing regimen	312 (58)	62 (38)	<0.0001
ICU stay, n (%)	197/534 (37)	82 (51)	0.002
Mechanical ventilation, n (%)	150/532 (28)	66 (41)	0.003
Final response to therapy, n (%)			0.003
Success	221/517 (43)	47/159 (30)	
Failure	296/517 (57)	112/159 (70)	
Mortality after IA diagnosis			
Overall 42-day mortality, n (%)	209/536 (39)	82 (51)	0.009
IA-associated 42-day mortality, n (%)	151/518 (29)	68/158 (43)	0.001
Overall 84-day mortality, n (%)	261/535 (49)	99 (61)	0.006
IA-associated 84-day mortality, n (%)	182/506 (36)	77/157 (49)	0.003

Abbreviations: IA, invasive aspergillosis. ICU, intensive care unit.

**Table 3:** Comparison of IA patients infected with *A. terreus* between different time periods.

Variables	1993–2003	2004–2013	2014–2023	*p*-Value for Trend
	(N = 104)	(N = 21)	(N = 37)	
Age (years), median (IQR)	49 (37–59)	59 (41–65)	64 (57–70)	<0.0001
Sex, male, n (%)	71 (68)	14 (67)	17 (46)	0.022
Diagnosis of IA, n (%)				0.003
Definite IA	32 (31)	2 (10)	3/36 (8)	
Probable IA	72 (69)	19 (90)	33/36 (92)	
Type of IA, n (%)				
Invasive pulmonary infection	90 (87)	20 (95)	34/36 (94)	0.14
Disseminated infection	7 (7)	0 (0)	0/36 (0)	0.06
Localized infection	6 (6)	0 (0)	2/36 (6)	0.79
Sinus infection	1 (1)	1 (5)	0/36 (0)	0.89
Type of hematological malignancy, n (%)				
Leukemia	88 (85)	16 (76)	17 (46)	<0.0001
Lymphoma	11 (11)	4 (19)	11 (30)	0.006
Myeloma	5 (5)	1 (5)	3 (8)	0.48
Transplant within 1 year before IA, n (%)	37 (36)	11 (52)	9/35 (26)	0.49
Type of transplant within 1 year before IA, n (%)				0.003
Autologous	2/37 (5)	1/10 (10)	4/9 (44)	
Allogeneic	35/37 (95)	9/10 (90)	5/9 (56)	
Graft-versus-host disease, n (%)	26 (25)	6/20 (30)	5/35 (14)	0.26
Neutropenia (ANC < 500) within 7 days of IA onset, n (%)	53 (51)	8/19 (42)	13/36 (36)	0.12
Primary antifungal therapy, n (%)				
Polyene-containing regimen	89 (86)	6 (29)	11 (30)	<0.0001
Echinocandin-containing regimen	15 (14)	9 (43)	13 (35)	0.003
Azole-containing regimen	24 (23)	16 (67)	24 (65)	<0.0001
ICU stay, n (%)	58 (56)	7 (33)	17 (46)	0.18
Mechanical ventilation, n (%)	46 (44)	6 (29)	14 (38)	0.37
Final response to therapy, n (%)				<0.0001
Success	13 (13)	13 (62)	21/34 (62)	
Failure	91 (88)	8 (38)	13/34 (38)	
Mortality after IA diagnosis				
Overall 42-day mortality, n (%)	66 (63)	5 (24)	11 (30)	<0.0001
IA-associated 42-day mortality, n (%)	61 (59)	4 (19)	3/33 (9)	<0.0001
Overall 84-day mortality, n (%)	81 (78)	5 (24)	13 (35)	<0.0001
IA-associated 84-day mortality, n (%)	69 (66)	4 (19)	4/32 (13)	<0.0001

Abbreviations: ANC, absolute neutrophil count per µL. IA, invasive aspergillosis. ICU, intensive care unit. IQR, interquartile range.

## Data Availability

The original contributions presented in this study are included in the article. Further inquiries can be directed to the corresponding author.
